# Supplementary material for: Flavor-switchable scaffold for cultured meat with enhanced aromatic properties
Source: Nat Commun. 2024 Jul 9;15:5450. doi: 10.1038/s41467-024-49521-5 (PMC11233498; doi:10.1038/s41467-024-49521-5)
Supplement: Supplementary file 1 — Supplementary Information [file 41467_2024_49521_MOESM1_ESM.pdf]

Supplementary Information

# Flavor-Switchable Scaffold for Cultured Meat with Enhanced Aromatic Properties

**Milae Lee[1†], Woojin Choi[1†], Jeong Min Lee[2], Seung Tae Lee[2], Won-Gun Koh[1], Jinkee Hong[1*]**

[†]These authors contributed equally to this work.

[1]Department of Chemical & Biomolecular Engineering, College of Engineering, Yonsei University, 50 Yonsei-ro, Seodaemun-gu, Seoul 03722, Republic of Korea

[2]Department of Applied Animal Life Science, Kangwon National University, 1 Kangwondaehak-gil, Chuncheon-si, Gangwon-do 24341, Republic of Korea

*Correspondence is addressed to J.H. (email: jinkee.hong@yonsei.ac.kr)

## Experimental method

### Bovine myoblast isolation

Primary bovine myoblasts were obtained from muscle tissues harvested from 29- to 31-month-old male or female Hanwoo cattle slaughtered at a local slaughterhouse (Kwell LPC, Hongcheon, Korea). All experimental procedures for animal slaughtering performed in this study complied with the Animal Care and Use Guidelines of Kangwon National University and were approved by the Institutional Animal Care and Use Committee (IACUC) of Kangwon National University (IACUC approval no. KW-220714-1).

For the myoblast isolation, the following process was performed. Firstly, the harvested muscle tissues were washed once in 70% ethanol and twice in 2% (v/v) antibiotic-antimycotic solution (AA; Welgene, LS203-01) diluted in Dulbecco's phosphate-buffered saline (DPBS; Welgene, LB001-02). Then, the muscle tissues were cut into small pieces, and digested using 0.2% (w/v) collagenase type II (Worthington Biochemical Corporation, LS004174) dissolved in high glucose-Dulbecco's Modified Eagle Medium (HG-DMEM; Welgene, LB001-05) supplemented with 1% (w/v) pronase (Calbiochem, 53702). Subsequently, the completely digested skeletal muscle tissues were re-suspended in HG-DMEM supplemented with 2% (v/v) heat-inactivated fetal bovine serum (FBS; Welgene, S101-01) for dissociation enzyme inactivation. After centrifuging the suspension at 1500 x g for 4 minuites, the pellets were re-suspended in red blood cell (RBC) lysis buffer (Sigma-Aldrich, 11814389001) for 10 minutes at room temperature to eliminate RBCs. Then, the RBC-free primary cells were filtered using a 100-um cell strainer (SPL, Korea), followed by filtration through a 70-um cell strainer (SPL). Finally, the muscle-derived primary cells descended by centrifuging at 1500 x g for 4 minutes, then re-suspended in the myoblast proliferation medium which is composed of HG-DMEM supplemented with 10% (v/v) FBS, 5 ng/mL basic fibroblast growth factor (bFGF; Peprotech,100-18B), and 1% (v/v) AA. To isolate myoblast s from the muscle-derived primary

cells, muscle-derived primary cells (5 x $10^5$) were seeded onto a 35-mm culture dish (SPL) and cultured in the myoblast proliferation medium. After 24 hours, non-adherent cells to the culture dishes were removed and the remaining adherent cells in the fresh myoblast proliferation medium were cultured at 37 °C in a humidified atmosphere of 5% $CO_2$ in air with medium exchange at 2-day intervals. When cell confluency reached 50-60%, the adherent cells were immunostained with the myoblast marker, MyoD (Santa Cruz, sc-377460 AF488), for myoblast characterization.

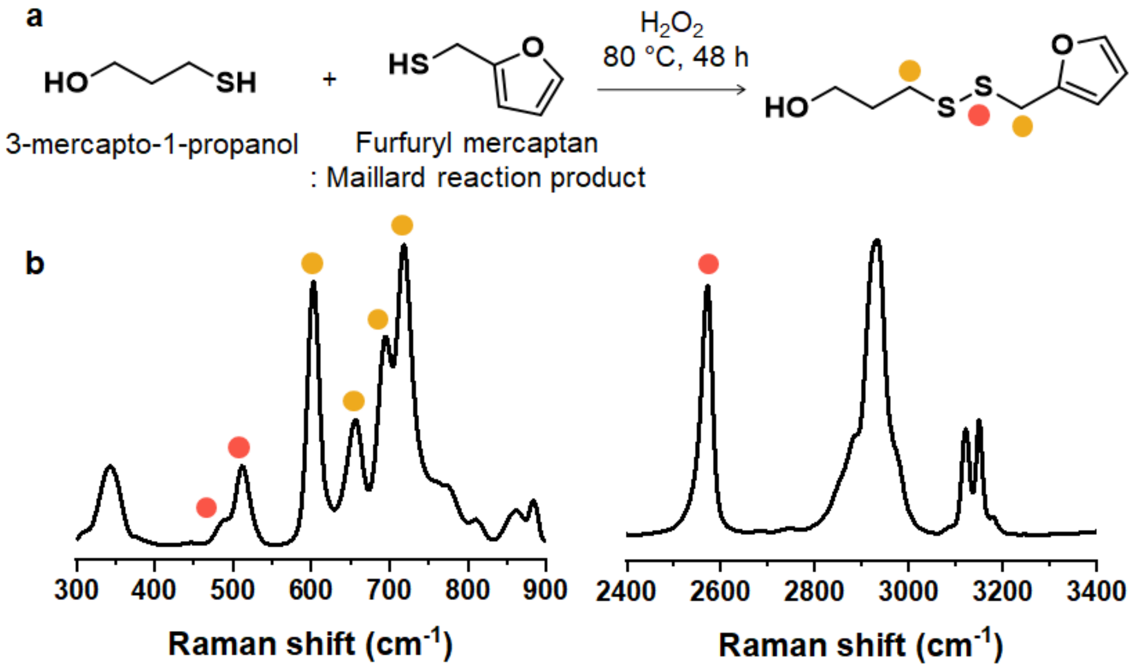


**Supplementary Fig. 1. Preparation of a Maillard reaction product involving disulfide bond.** **a** Disulfide bond formation of representative Maillard reaction product, furfuryl mercaptan. The hydrogen peroxide of 2.56 mmol was employed to induce the disulfide linkage between 11.6 mmol 3-mercapto-1-propanol and 11.6 mmol furfuryl mercaptan[1]. **b** Raman spectra of disulfide bond-introduced Maillard reaction product. The Raman signals at 486 $cm^{-1}$, 515 $cm^{-1}$, and 2,570 $cm^{-1}$ correspond to the disulfide bonds[2]. Source data are provided as a Source Data file.

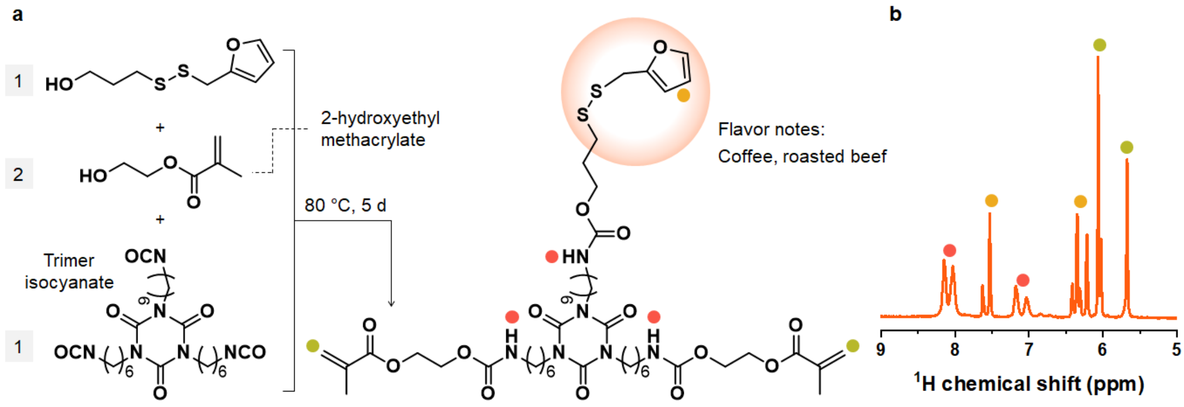


**Supplementary Fig. 2. Preparation of a switchable flavor compound (SFC). a** Disulfide bond-introduced Maillard reaction product, 2-hydroxyethyl methacrylate, and trimer-isocyanate were reacted in 1:2:1 molar ratio at 80 °C for 5 d to synthesize SFC[3]. **b** $^{1}$H nuclear magnetic resonance (NMR) spectra of SFC. The signals of urethane bond (red circles), acrylate (green circles), and furan (yellow circles) indicate the formation of SFC. Source data are provided as a Source Data file.

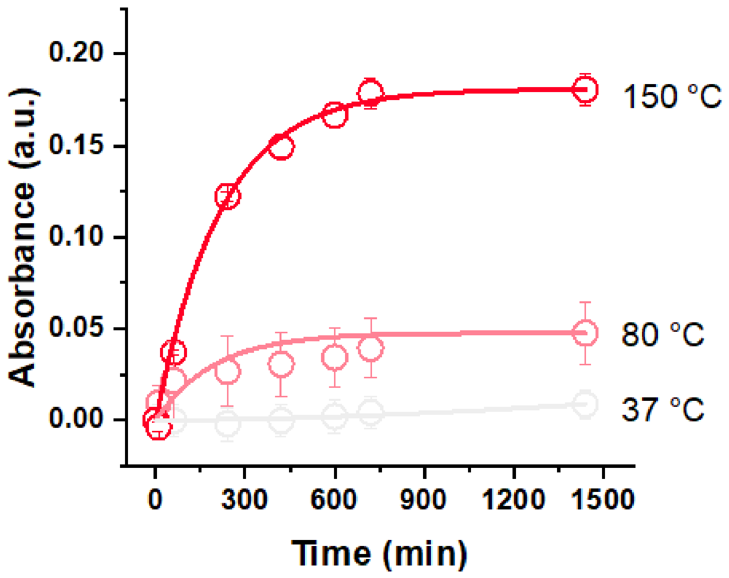


**Supplementary Fig. 3. Thermal responsivity of SFC.** The ultraviolet-visible spectra of SFC was measured within 270-400 nm wavelength following the heating at 37 ℃, 80 ℃, and 150 ℃ during 24 h. The mobility of Maillard reaction product was suggested by the furan-related absorbance signal at 335 nm. Source data are provided as a Source Data file.

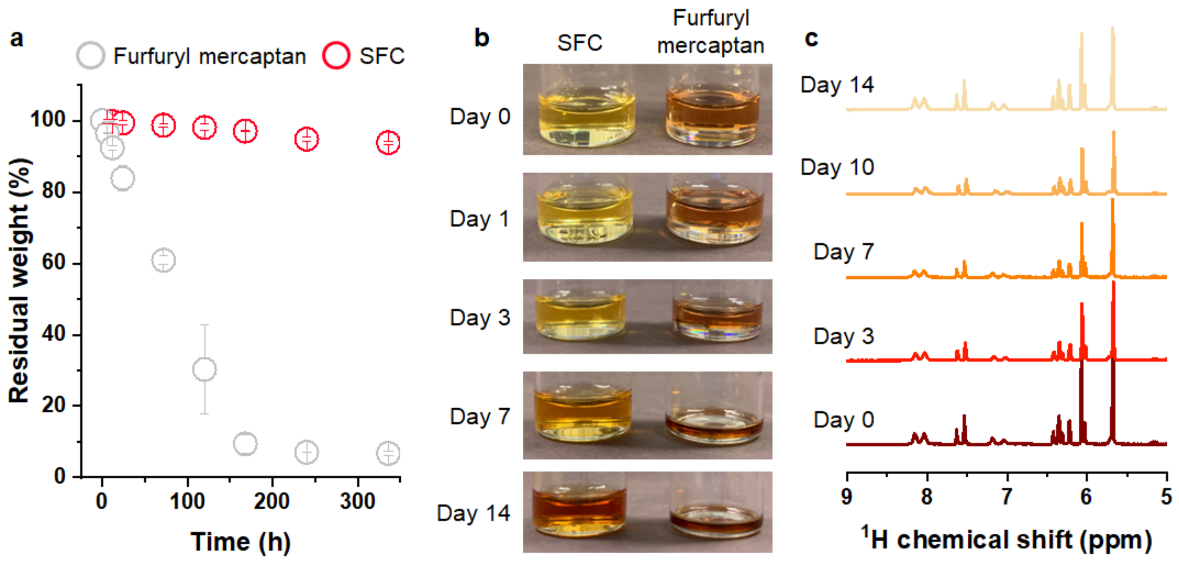


**Supplementary Fig. 4. Stability of SFC. a** Residual weight and **b** volume changes of pure furfuryl mercaptan and SFC during 14 days of 37 °C incubation at the open system. Initial volume of furfuryl mercaptan and SFC was 1 mL. **c** $^{1}H$ NMR spectra of SFC during 14 days of 37 °C incubation. No significant spectral changes were observed. Source data are provided as a Source Data file.

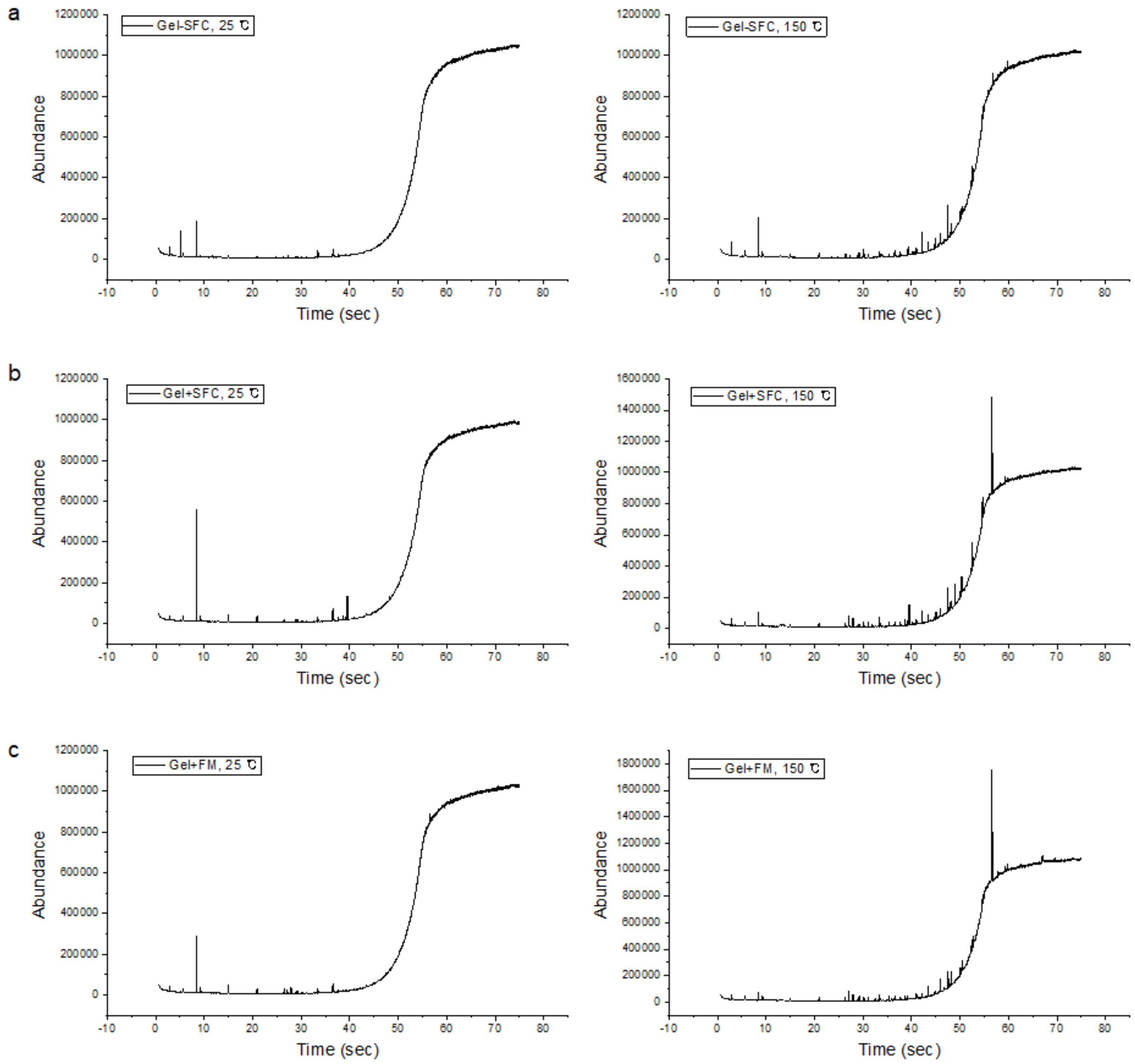


**Supplementary Fig. 5. GC-MS results of the hydrogels in Fig. 2d. a** Chromatograms of the hydrogel without SFC (Gel-SFC) at 25 °C and at 150 °C **b** Chromatograms of the hydrogel with SFC (Gel+SFC) at 25 °C and at 150 °C **c** Chromatograms of the hydrogel mixed with furfuryl mercaptan (Gel+FM) at 25 °C and at 150 °C. Source data are provided as a Source Data file.

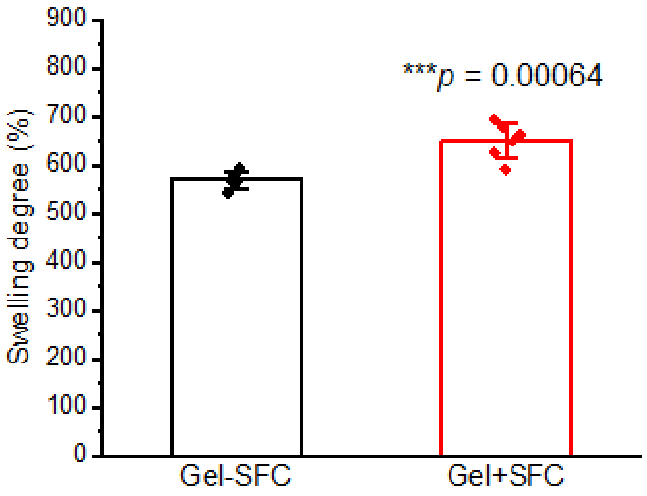


**Supplementary Fig. 6. Swelling degree of Gel-SFC and Gel+SFC measured after immersing each scaffold in culture media for 24 hours at 37 °C (mean ± SD, *n* = 6 independent experiments, One-way ANOVA with Tukey method).** The swelling degree was calculated by the following equation.

$$\text{Swelling degree (\%)} = \frac{(\text{Weight of swollen scaffold} - \text{Weight of lyophilized scaffold}) * 100}{\text{Weight of lyophilized scaffold}}$$

Source data are provided as a Source Data file.

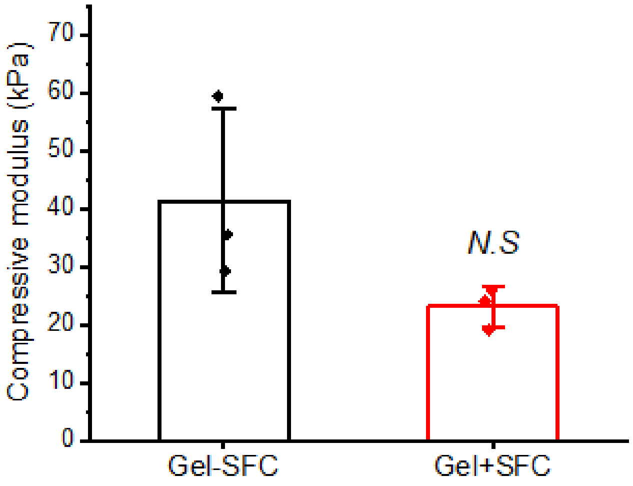


**Supplementary Fig. 7. Stiffness of Gel-SFC and Gel+SFC measured after immersing each scaffold in culture media for 24 hours at 37 °C (mean ± SD, *n* = 3 independent experiments, One-way ANOVA with Tukey method).** Source data are provided as a Source Data file.

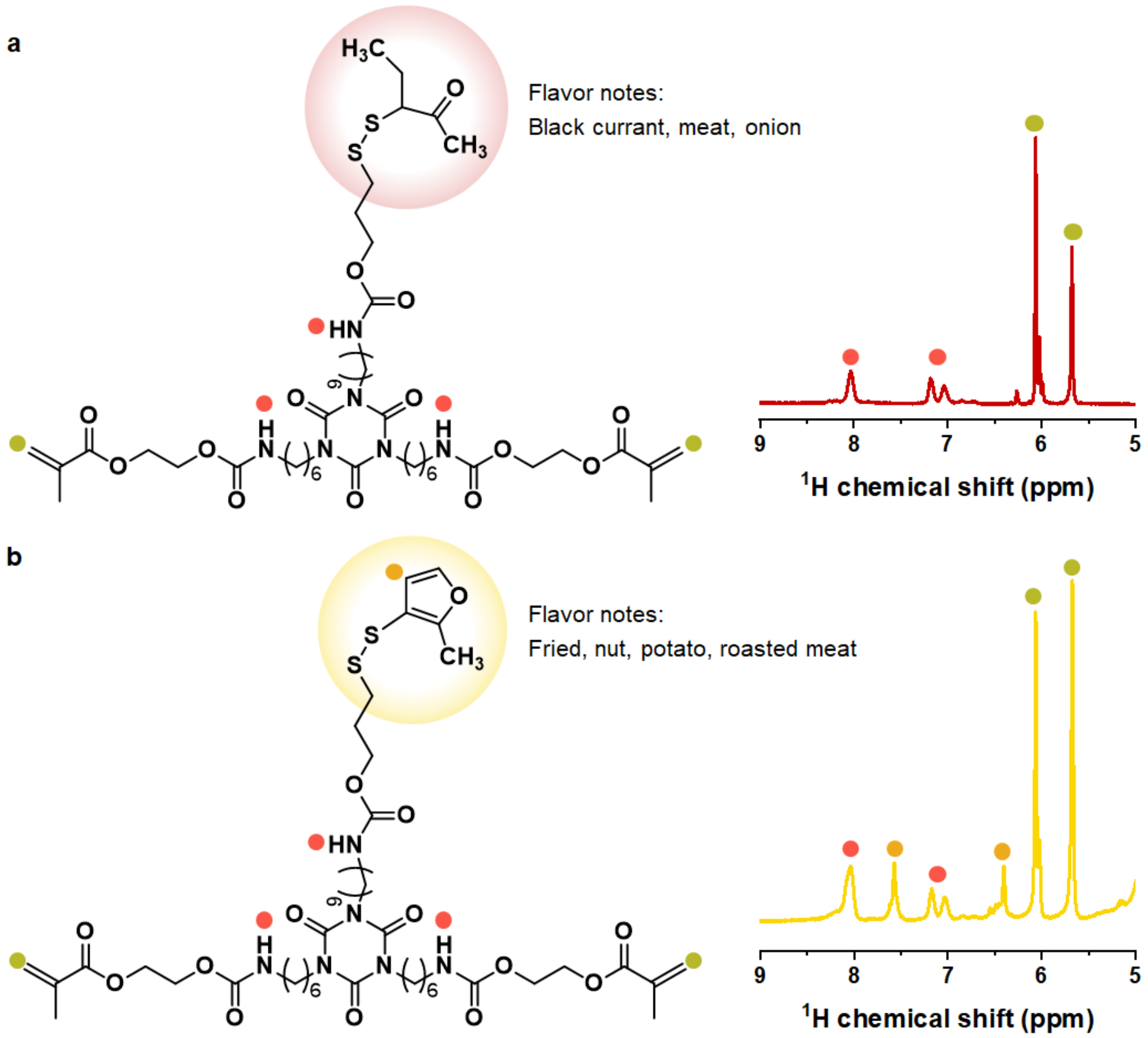


**Supplementary Fig. 8. Chemical structure of flavor variated switchable flavor compounds (SFCV). a** Switchable flavor compound (SFC) with the flavors of black currant, meat, and onion was prepared using 3-mercapto-2-pentanone. **b** SFC with the flavors of nut, potato, roasted meat was also prepared based on 2-methyl-3-furanthiol. **a-b** Same protocols of disulfide bond introduction and urethane reaction were conducted. Source data are provided as a Source Data file.

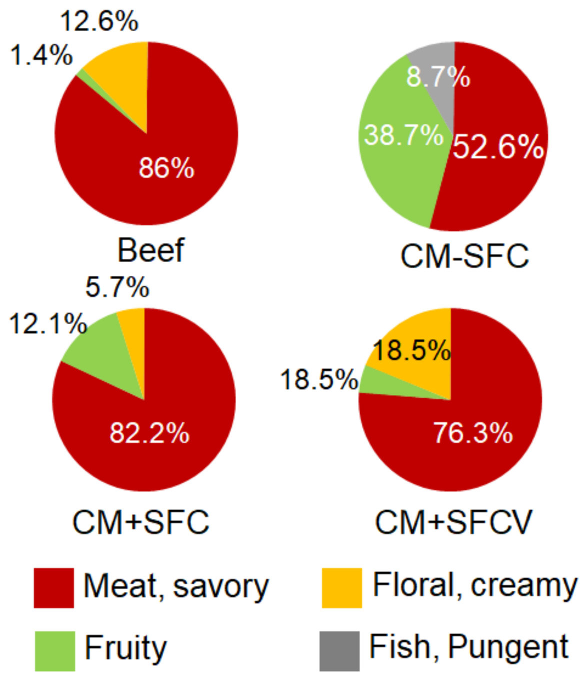


**Supplementary Fig. 9. Flavor profiles of beef, cultured meat without switchable flavor compound (CM-SFC), cultured meat with SFC (CM+SFC), and cultured meat with flavor variated SFC (CM+SFCV).** Source data are provided as a Source Data file.

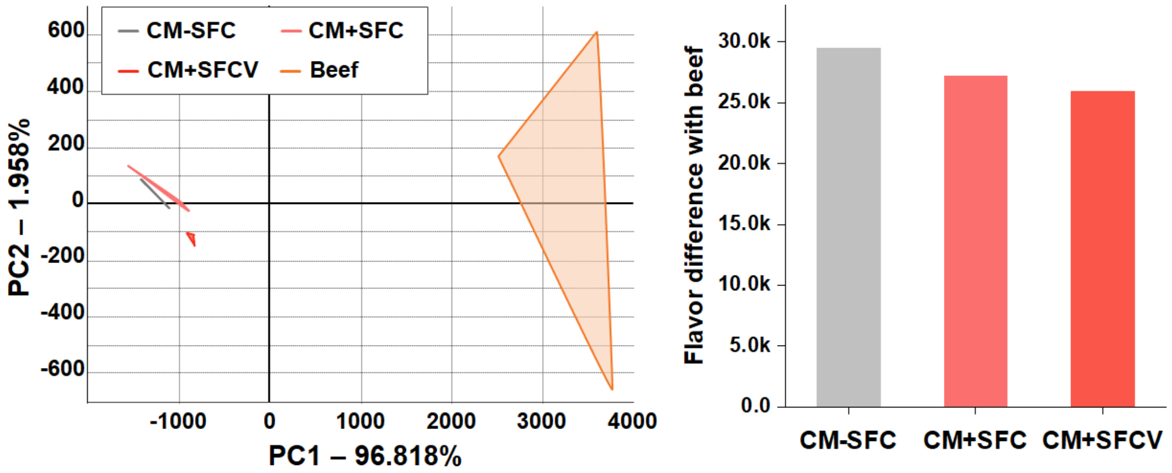


**Supplementary Fig. 10. Principal component analysis (PCA) of the flavor compounds detected from beef, cultured meat without switchable flavor compound (CM-SFC), cultured meat with SFC (CM+SFC), and cultured meat flavor variated SFC (CM+SFCV)** (Discrimination index = 82, *n* = 3). Source data are provided as a Source Data file.

| Scaffold type | Temperature | Flavor description | Detected flavor compound (IUPAC name) |
|---|---|---|---|
| Gel-SFC | 25 °C | Meaty, Savory | Not detected |
| | | Almond, Roasted bread | Not detected |
| | | Floral, Cheese, Fat | Not detected |
| | | Fishy, Pungent, Sour | Acetone |
| | 150 °C | Meaty, Savory | Not detected |
| | | Almond, Roasted bread | Benzaldehyde, Benzyl alcohol (Phenylmethanol) |
| | | Floral, Cheese, Fat | Nonanoic acid, Octanoic acid |
| | | Fishy, Pungent, Sour | Hexanoic acid |
| Gel+FM | 25 °C | Meaty, Savory | Furfuryl mercaptan (furan-2-ylmethanethiol), Furfural (furan-2-carbaldehyde) |
| | | Almond, Roasted bread | Not detected |
| | | Floral, Cheese, Fat | Not detected |
| | | Fishy, Pungent, Sour | Not detected |
| | 150 °C | Meaty, Savory | Furfuryl mercaptan (furan-2-ylmethanethiol), Furfural (furan-2-carbaldehyde), 2,2-(dithiodimethylene)difuran |
| | | Almond, Roasted bread | Not detected |
| | | Floral, Cheese, Fat | Nonanoic acid |
| | | Fishy, Pungent, Sour | Not detected |
| Gel+SFC | 25 °C | Meaty, Savory | Not detected |
| | | Almond, Roasted bread | Not detected |
| | | Floral, Cheese, Fat | Not detected |
| | | Fishy, Pungent, Sour | Not detected |
| | 150 °C | Meaty, Savory | Furfuryl mercaptan (furan-2-ylmethanethiol), Furfural (furan-2-carbaldehyde), 2,2-(dithiodimethylene)difuran |
| | | Almond, Roasted bread | Benzaldehyde, Benzyl alcohol (Phenylmethanol) |
| | | Floral, Cheese, Fat | Nonanoic acid, Heptanoic acid, Octanoic acid |
| | | Fishy, Pungent, Sour | Not detected |

**Supplementary Table 1. Flavor compounds detected from the hydrogel without switchable flavor compound (Gel-SFC), hydrogel with SFC (Gel+SFC), and hydrogel mixed with pure furfuryl mercaptan (Gel+FM) depending on the temperature.**

| Cultured meat type | Flavor description | Detected flavor compound |
|---|---|---|
| CM-SFC | Meaty, savory | Methanethiol, 2-methylthiophene |
| | Floral, Creamy | Not detected |
| | Fruity | Carbon disulfide, n-butanol, Methyl but-2-enoate |
| | Fish, Pungent | Trimethylamine |
| CM+SFC | Meaty, savory | Methanethiol |
| | Floral, Creamy | Butane-2,3-dione, Pent-1-en-3-ol |
| | Fruity | Carbon disulfide |
| | Fish, Pungent | Not detected |
| CM+SFCV | Meaty, savory | Methanethiol, 2-methylthiophene |
| | Floral, Creamy | Pentanoic acid |
| | Fruity | Heptyl pentanoate |
| | Fish, Pungent | Not detected |

**Supplementary Table 2. Flavor compounds detected from the cultured meat without switchable flavor compound (CM-SFC), cultured meat with SFC (CM+SFC), and cultured meat with flavor variated SFC (CM+SFCV) by electronic nose.**

| Reagent name | Company | Catalog number |
|---|---|---|
| 3-mercapto-1-propanol | TCI-SEJIN CI | M1206 |
| Furfuryl mercaptan | TCI-SEJIN CI | F0077 |
| 3-mercapto-2-pentanone | TCI-SEJIN CI | M2026 |
| 2-methyl-3-furanthiol | TCI-SEJIN CI | M1847 |
| Hydrogen peroxide | Sigma Aldrich | 516813 |
| Hexamethylene diisocyanate isocyanurate trimer | BLD Pharm | 3779-63-2 |
| 2-hydroxyethyl methacrylate | Sigma Aldrich | 128635 |
| Propylene caebonate | Sigma Aldrich | 110264 |
| Dimethyl sulfoxide-d6 | Sigma Aldrich | 151874 |
| Fish gelatin | GELTECH | - |
| Methacrylic anhydride | Sigma Aldrich | 276685 |
| 2-hydroxy-4-(2-hydroxyethoxy)-2-methylpropiophenone | Sigma Aldrich | 410896 |
| High-glucose Dulbecco's modified Eagle medium (HG-DMEM) | Thermo Fisher Scientific | LB001-05 |
| Heat-inactivated fetal bovine serum (FBS) | Welgene | S101-01 |
| Penicillin-streptomycin-glutamine (PS) | Gibco® Life Technologies | 10378016 |
| Basic fibroblast growth factor (bFGF) | Peprotech | 100-18B |
| Phosphate buffer saline (PBS) | Gibco® Life Technologies | 10010031 |
| Trypsin-EDTA | Welgene | LS015-10 |
| Horse serum | Thermo Fischer Scientific | 26050088 |
| 70% Ethanol | DAEJUNG | 4018-4410 |
| D-Plus™ CCK cell viability assay kit | Dongin LS | CCK-3000 |
| Formalin solution, neutral buffered, 10% | Sigma Aldrich | HT5012 |
| Bovine serum albumin | Sigma Aldrich | A3311 |
| Triton™ X-100 solution | Sigma Aldrich | 93443 |
| Alexa Fluor 488™ phalloidin | Thermo Fisher Scientific | A12379 |
| DAPI | Thermo Fisher Scientific | D9542 |
| MF 20 (ID: AB2147781) | DSHB | AB2147781 |
| Donkey anti-mouse Alexa Flour 594 | Thermo Fisher Scientific | A21203 |
| Bovine myosin-1 (MYH1) ELISA kit | MyBioSource | MBS7229767 |

**Supplementary Table 3. List of the reagents used in the method section.**

| Product | Company | Catalog number |
|---|---|---|
| 12-14 kDa membrane | Thermo Fisher | 08667E |
| 24-well plate | SPL | 31024 |
| Carboxen/polydimethylsiloxane/divinylbenzene (CAR/PDMS/DVB) fiber | Sigma Aldrich | 57348-U |
| DB-WAX column | Agilent | 123-7063 |
| TPP® tissue culture dishes | Sigma Aldrich | 93100 |
| MXT-5 GC metal capillary column | Restek | 70223 |
| MXT-1701 GC metal capillary column | Restek | 72023 |

**Supplementary Table 4. List of the products used in the method section.**

## Supplementary references


1 García-Santamarina, S., Boronat, S. & Hidalgo, E. Reversible cysteine oxidation in hydrogen peroxide sensing and signal transduction. *Biochemistry* **53**, 2560-2580 (2014).

2 Li, J. *et al.* Copolymer of poly (ethylene glycol) and poly (L-lysine) grafting polyethylenimine through a reducible disulfide linkage for siRNA delivery. *Nanoscale* **6**, 1732-1740 (2014).

3 Choi, W. *et al.* Templated Assembly of Silk Fibroin for a Bio-Feedstock-Derived Heart Valve Leaflet. *Advanced Functional Materials*, 2307106 (2023).
