# Supplementary material for: Flavor-switchable scaffold for cultured meat with enhanced aromatic properties
Source: Nat Commun. 2024 Jul 9;15:5450. doi: 10.1038/s41467-024-49521-5 (PMC11233498; doi:10.1038/s41467-024-49521-5)
Supplement: Supplementary file 2 — Reporting Summary [file 41467_2024_49521_MOESM2_ESM.pdf]

nature portfolio

Corresponding author(s): Jinkee Hong

Last updated by author(s): May 30, 2024

Reporting Summary

Nature Portfolio wishes to improve the reproducibility of the work that we publish. This form provides structure for consistency and transparency in reporting. For further information on Nature Portfolio policies, see our Editorial Policies and the Editorial Policy Checklist.

Statistics

For all statistical analyses, confirm that the following items are present in the figure legend, table legend, main text, or Methods section.

n/a	Confirmed	
☐	☒	The exact sample size (*n*) for each experimental group/condition, given as a discrete number and unit of measurement
☐	☒	A statement on whether measurements were taken from distinct samples or whether the same sample was measured repeatedly
☐	☒	The statistical test(s) used AND whether they are one- or two-sided<br>*Only common tests should be described solely by name; describe more complex techniques in the Methods section.*
☐	☒	A description of all covariates tested
☐	☒	A description of any assumptions or corrections, such as tests of normality and adjustment for multiple comparisons
☐	☒	A full description of the statistical parameters including central tendency (e.g. means) or other basic estimates (e.g. regression coefficient) AND variation (e.g. standard deviation) or associated estimates of uncertainty (e.g. confidence intervals)
☐	☒	For null hypothesis testing, the test statistic (e.g. *F*, *t*, *r*) with confidence intervals, effect sizes, degrees of freedom and *P* value noted<br>*Give P values as exact values whenever suitable.*
☒	☐	For Bayesian analysis, information on the choice of priors and Markov chain Monte Carlo settings
☒	☐	For hierarchical and complex designs, identification of the appropriate level for tests and full reporting of outcomes
☒	☐	Estimates of effect sizes (e.g. Cohen's *d*, Pearson's *r*), indicating how they were calculated

Our web collection on statistics for biologists contains articles on many of the points above.

Software and code

Policy information about availability of computer code

Data collection	Chem Draw Ultra 12.0, Origin Pro (version 2018), ZEISS ZEN (version 3.2), Image J software (version 1.52a, National Institute of Health, Bethesda, MD, USA), and Alpha Soft software (Alpha MOS, France) were used.
Data analysis	Origin Pro (version 2018) was used for statistical analysis. Statistical analysis was performed with the significance level of 0.05 using One-way ANOVA with Tukey method. All experiments were repeated three times independently.

For manuscripts utilizing custom algorithms or software that are central to the research but not yet described in published literature, software must be made available to editors and reviewers. We strongly encourage code deposition in a community repository (e.g. GitHub). See the Nature Portfolio guidelines for submitting code & software for further information.

Data

Policy information about availability of data

All manuscripts must include a data availability statement. This statement should provide the following information, where applicable:

- Accession codes, unique identifiers, or web links for publicly available datasets
- A description of any restrictions on data availability
- For clinical datasets or third party data, please ensure that the statement adheres to our policy

All data supporting the findings of this study are available within the paper and its Supplementary Information. Source data are also provided with this paper.

Research involving human participants, their data, or biological material

Policy information about studies with human participants or human data. See also policy information about sex, gender (identity/presentation), and sexual orientation and race, ethnicity and racism.

Reporting on sex and gender	There was no human research participants
Reporting on race, ethnicity, or other socially relevant groupings	There was no human research participants
Population characteristics	There was no human research participants
Recruitment	There was no human research participants
Ethics oversight	There was no human research participants

Note that full information on the approval of the study protocol must also be provided in the manuscript.

Field-specific reporting

Please select the one below that is the best fit for your research. If you are not sure, read the appropriate sections before making your selection.

[x] Life sciences [ ] Behavioural & social sciences [ ] Ecological, evolutionary & environmental sciences

For a reference copy of the document with all sections, see nature.com/documents/nr-reporting-summary-flat.pdf

Life sciences study design

All studies must disclose on these points even when the disclosure is negative.

Sample size	No sample size calculation was performed before the experiments. Sample size was determined to be 3 per each group to obtain statistically meaningful conclusion such as differences or similarities between groups.
Data exclusions	No data were excluded from the analyses
Replication	All the key experiments were replicated at least three times
Randomization	All samples were randomly allocated into experimental groups
Blinding	Blinding did not apply to this study as all samples were processed with identical conditions

Reporting for specific materials, systems and methods

We require information from authors about some types of materials, experimental systems and methods used in many studies. Here, indicate whether each material, system or method listed is relevant to your study. If you are not sure if a list item applies to your research, read the appropriate section before selecting a response.

Materials & experimental systems

n/a	Involved in the study	
[ ]	[x]	Antibodies
[x]	[ ]	Eukaryotic cell lines
[x]	[ ]	Palaeontology and archaeology
[x]	[ ]	Animals and other organisms
[x]	[ ]	Clinical data
[x]	[ ]	Dual use research of concern
[x]	[ ]	Plants

Methods

n/a	Involved in the study	
[x]	[ ]	ChIP-seq
[x]	[ ]	Flow cytometry
[x]	[ ]	MRI-based neuroimaging

Antibodies

Antibodies used	For immunostaining, first antibody was used:<br>Myosin heavy chain antibody (MF 20, DSHB, ID: AB2147781) - diluted 100 folds when used.<br>Secondary antibody included in this study:<br>Donkey anti-Mouse IgG (H+L) Highly Cross-Adsorbed Secondary Antibody, Alexa Fluor 594 (A-21203, Thermo Fisher) - used in 0.005 mg/mL concentration.

	Alexa Fluor 488TM phalloidin (A12379, Thermo Fisher) - used in 0.165 uM concentration.
Validation	Myosin heavy chain antibody (https://dshb.biology.uiowa.edu/MF-20)

Plants

Seed stocks	N/A
Novel plant genotypes	N/A
Authentication	N/A
